# Light Regulation of Axillary Bud Outgrowth Along Plant Axes: An Overview of the Roles of Sugars and Hormones

**DOI:** 10.3389/fpls.2019.01296

**Published:** 2019-10-18

**Authors:** Anne Schneider, Christophe Godin, Frédéric Boudon, Sabine Demotes-Mainard, Soulaiman Sakr, Jessica Bertheloot

**Affiliations:** ^1^IRHS, INRA, Agrocampus-Ouest, Université d’Angers, SFR 4207 QuaSaV, Beaucouzé, France; ^2^Laboratoire Reproduction et Développement des Plantes, Univ Lyon, ENS de Lyon, UCB Lyon 1, CNRS, INRA, INRIA, Lyon, France; ^3^CIRAD, UMR AGAP & Univ. Montpellier, Montpellier, France

**Keywords:** light, hormones, sugar, bud outgrowth, branching, apical dominance, cytokinins, R:FR

## Abstract

Apical dominance, the process by which the growing apical zone of the shoot inhibits bud outgrowth, involves an intricate network of several signals in the shoot. Auxin originating from plant apical region inhibits bud outgrowth indirectly. This inhibition is in particular mediated by cytokinins and strigolactones, which move from the stem to the bud and that respectively stimulate and repress bud outgrowth. The action of this hormonal network is itself modulated by sugar levels as competition for sugars, caused by the growing apical sugar sink, may deprive buds from sugars and prevents bud outgrowth partly by their signaling role. In this review, we analyze recent findings on the interaction between light, in terms of quantity and quality, and apical dominance regulation. Depending on growth conditions, light may trigger different pathways of the apical dominance regulatory network. Studies pinpoint to the key role of shoot-located cytokinin synthesis for light intensity and abscisic acid synthesis in the bud for R:FR in the regulation of bud outgrowth by light. Our analysis provides three major research lines to get a more comprehensive understanding of light effects on bud outgrowth. This would undoubtedly benefit from the use of computer modeling associated with experimental observations to deal with a regulatory system that involves several interacting signals, feedbacks, and quantitative effects.

## Introduction

As sessile organisms, plants have to adapt to their growth environment. One important way is to adapt their branching architecture, above and below grounds, to accommodate endogenous (e.g., water and carbon status) and exogenous (light, space) constraints. In this process, branching regulation plays a crucial role as it defines strategies whereby plants colonize the underground and aerial spaces. Different environmental factors have been shown to impact this process, such as mineral or water supply to the roots, light, or temperature (Bouguyon et al., 2012; de Jong et al., 2014; Pierik and Testerink, 2014; Li-Marchetti et al., 2015). In the past two decades, due to spectacular advances in biotechnology, imaging, molecular biology, and computational modeling, major breakthroughs have been made in the understanding of the physiological regulation of branching of aerial axes. In particular, the veil on the key mechanisms whereby light regulates aerial branching on plant axes has been partly lifted.

During growth, apical meristems of plant axes produce sequences of phytomers. One phytomer is composed of an internode with its axillary leaf and one or several axillary buds. Once initiated, axillary buds themselves may in turn enter growth immediately (sylleptic buds), or they can remain latent (proleptic buds) until some external event to the buds triggers their outgrowth (Lang et al., 1987; Kieffer et al., 1998; Barthelemy and Caraglio, 2007). This latter two-phase strategy is very frequent in both annual or perennial plants and has been shown to result from the dominance of the growing apex over its axillary meristems. This phenomenon, called apical dominance, offers plants the possibility to develop in a parsimonious way while preserving the possibility of branching to adapt their development to changing physiological or environmental contexts (Cline, 1994).

Light in particular has been recognized as a major modulator of the expression of apical dominance for decades. For example, increasing light intensity in photosynthetically active radiation (PAR) often results in an increase of the total number of lateral branches that develop on a given axis, thus reducing apical dominance (Mitchell, 1953; Su et al., 2011; Demotes-Mainard et al., 2013; Leduc et al., 2014). Likewise, a change in light quality, such as a high red-to-far-red wavelength ratio (R:FR) due to the use of red LEDs in a greenhouse or to gaps in a canopy, often leads to an increase of the number of outgrowing branches (Demotes-Mainard et al., 2016). In principle, these modulations may result from either an increase of the total number of primary nodes or from the probability for a bud to grow out. Light may affect both processes, resulting in significant modulations of branching intensity and plant architecture. Finally, light may also, in a more subtle way, affect the time taken by axillary buds to enter into growth (Bos and Neuteboom, 1998; Gautier et al., 1999; Evers et al., 2006; Demotes-Mainard et al., 2016).

The nature of the physiological or biophysical mechanisms whereby light interacts with the process of apical dominance and participates to releasing axillary bud latency is still largely elusive. A better understanding of these mechanisms requires identifying how light interacts with the physiological mechanisms regulating apical dominance. Two major putative mechanisms of apical dominance have been debated in the literature over the last decades. First, it has been experimentally shown on a variety of plant species that apical dominance is mediated by the plant hormone auxin, produced at the growing apex, and transported downward through the vascular tissues of the stem (Thimann and Skoog, 1933; Cline, 1996; Ongaro and Leyser, 2008). In this view, the leading apex continuously produces auxin, which reaches bud neighborhood through basipetal transport, and controls bud outgrowth indirectly. Two main signaling cascades have been identified (Domagalska and Leyser, 2011): (i) auxin in the stem controls the production of two other hormones, cytokinins and strigolactones, that move into the bud to control its outgrowth (second messenger theory); and (ii) auxin transport itself prevents auxin export out of the bud, a process necessary for bud outgrowth (canalization theory). These signaling cascades inhibit bud outgrowth as long as the main apex keeps producing auxin. This signaling hypothesis has long been challenged by a second hypothesis based on competition for resources (Luquet et al., 2006). This alternative view is based on the idea that during growth, plant organs compete for nutrients, and growing organs divert the nutrient resources from the freshly created buds. Deprived of resources, these buds remain latent as long as the main apex continues to grow. It was recently suggested that both hypotheses could be coupled in the regulation of bud outgrowth (Barbier et al., 2015; Barbier et al., 2019; Bertheloot et al., 2019).

In this review, we analyze how the effect of light on bud outgrowth has been interpreted in the context of the two main paradigms thought to be at the origin of apical dominance (which excludes the question of endodormancy in perennial plants). While previous review mainly focused on light effects in the vicinity of the bud (Leduc et al., 2014), this review aims to analyze how current knowledge from physiological and modeling studies helps to get a comprehensive understanding of light effects at the plant level. We start by a brief description of the main endogenous regulators of apical dominance, and their interaction and modulation at the plant scale. The hormonal regulation is described in a first section, while the regulation by the competition for nutrients is described in a second section. Then, we analyze the current knowledge about how light interacts with the previously identified endogenous network, including hormones and nutrients. We finally discuss the major gaps in the building of a comprehensive understanding of light-mediated bud outgrowth regulation and stress the potential complexity of the regulatory network, involving interactions between several regulators, dose-dependent effects, and feedback processes. We discuss why further detailed and quantitative analysis of this interaction will most probably require combining experimental and computational modeling approaches.

## Hormonal Regulation of Bud Outgrowth

### Regulation of Apical Dominance in the Shoot

#### The Regulators of Apical Dominance: Auxin, Cytokinins, and Strigolactones

Auxin, a plant hormone produced in the apical region and transported downwards through the stem, has long been considered as the orchestrator of apical dominance in plants (Thimann and Skoog, 1933; Thimann and Skoog, 1934; Rinne et al., 1993; Ljung et al., 2001; Ongaro and Leyser, 2008; Teichmann and Muhr, 2015). While decapitation of the growing shoot tip promotes bud outgrowth, exogenous auxin applied to the decapitated shoot tip usually restores bud outgrowth inhibition (Thimann and Skoog, 1933; Thimann and Skoog, 1934; Cline, 1996). Furthermore, plants with reduced or increased auxin signaling/level display increased or reduced branching levels, respectively (Romano et al., 1991; Booker et al., 2003).

Auxin acts in an interconnected way with two other hormones, cytokinins (CKs) and strigolactones (SLs). CKs act as shoot-branching inducers that have an antagonistic effect to auxin on bud outgrowth (Wickson and Thimann, 1958; Sachs and Thimann, 1967; Shimizu-Sato et al., 2009; Mueller and Leyser, 2011). SLs act as shoot-branching repressors and enhance the inhibiting effect of auxin on branching (Beveridge, 2000; Beveridge, 2006 for reviews; Gomez-Roldan et al., 2008; Umehara et al., 2008; Crawford et al., 2010). While CKs and SLs are synthesized in both shoots and roots, only CKs can move through both the xylem sap (tZ-type) and the phloem sap (iP-type) (Bangerth, 1994; Mader et al., 2003; Kudo et al., 2010; Mueller and Leyser, 2011). SLs move primarily acropetally through the transpiration stream of the xylem sap, while their receptor—protein D14—is transported through the phloem to axillary buds in rice (Kohlen et al., 2011; Kameoka et al., 2016).

Auxin cannot enter buds (Prasad et al., 1993; Booker et al., 2003) and indirectly inhibits bud outgrowth. Several years of experiments have demonstrated that auxin acts through at least two non-exclusive mechanisms at the nodal segment and shoot scales, respectively (see Domagalska and Leyser, 2011; Rameau et al., 2015).

#### The Regulating System At the Scale of the Nodal Segment Adjacent to the Bud

In a theory known as “the second messenger theory,” auxin in the nodal segment adjacent to the bud down-regulates CKs and up-regulates SLs, which are both supposed to migrate into the adjacent bud to control its outgrowth. The direct action of CKs and SLs in buds is supported by exogenous application of CKs and SLs on buds, which stimulated and inhibited their outgrowth, respectively (Sachs and Thimann, 1967; Gomez-Roldan et al., 2008; Dun et al., 2012). Furthermore, CK biosynthesis was rapidly enhanced in the nodal stem segment, and the CK content increased in the bud in response to auxin depletion, and these behaviors were prevented by exogenous auxin supply (Nordstrom et al., 2004; Tanaka et al., 2006; Liu et al., 2011; Li et al., 2018). By contrast, auxin depletion resulted in a rapid repression of SL biosynthesis-related genes in the stem, a behavior prevented by exogenous auxin application (Foo et al., 2005; Zou et al., 2006; Hayward et al., 2009).

The integration of the two antagonistic regulators CKs and SLs is at least partly mediated by the TCP transcriptional regulator TEOSINTE1/BRANCHED1 (*TB1*/*BRC1*) in the bud (for reviews Rameau et al., 2015; Wang et al., 2019). BRC1 locally inhibits bud outgrowth, and its transcript level can be downregulated by CKs and upregulated by SLs. However, the expression level of *OsTB1/FC1 (Oryza sativum Teosinte1/Fine Culm1*) in rice was insensitive to SLs (Minakuchi et al., 2010; Guan et al., 2012), and CKs promoted bud activation in pea *brc1* mutants (Braun et al., 2012). These results indicate that integration of CKs and SLs also involves a BRC1-independent pathway.

#### The Systemic Regulation System

In the “auxin canalization” theory, auxin transport in the stem is a systemic signal that prevents auxin export out of buds independently of any messengers relaying auxin signaling from the stem to the bud, and auxin export out of buds is necessary for their outgrowth. This theory relies on the observed tight correlation between bud outgrowth and auxin export out of the bud (Li and Bangerth, 1999; Bennett et al., 2006; Balla et al., 2011). As initially proposed by Sachs (1981) in the context of vascular strand differentiation, lateral auxin flow from the buds to the stem could be inhibited by the process of auxin canalization in the main stem, whereby the auxin flux upregulates and polarizes its own transport in one direction. From the 2000s, the identification of PIN auxin efflux carriers and visualization techniques based on PIN immunolocalization demonstrated the existence of a positive feedback between the auxin flow and its own transport. PIN polar targeting at the level of cell plasma membranes directs auxin flow, and this process is positively feedback-regulated by auxin itself (Sauer et al., 2006; Wisniewska et al., 2006). Introduction of such a feedback in a computer model confirmed the plausibility of the canalization theory. Prusinkiewicz et al. (2009) demonstrated through simulations that this feedback led to high auxin fluxes in the main stem, which may in turn prevent any lateral auxin flux from axillary buds. By stating that buds cannot enter sustained growth if they do not export their own auxin, auxin canalization in the main stem may thus explain bud inhibition during apical dominance. In this process, the directionality of canalization is determined by the auxin source that becomes active first (the apical one during apical dominance). Such a model also simulated several branching phenotypes observed in *Arabidopsis* mutants for auxin homeostasis or transport.

The discovery that SLs dampen polar auxin transport in the stem by down-regulating PIN accumulation in xylem parenchyma cells and triggering the rapid removal of PIN from the plasma membrane further confirmed the plausibility of the canalization theory (Bennett et al., 2006; Crawford et al., 2010; Xu et al., 2015; Li et al., 2018). A computational model in which the action of SLs is represented as an increase in the rate of removal of the auxin export protein—PIN—from the plasma membrane reproduced auxin transport and shoot branching phenotypes observed in various mutant combinations and SL treatments, including the counterintuitive ability of SLs to promote or inhibit shoot branching depending on the auxin transport status of the plant (Shinohara et al., 2013). Furthermore, exogenous supply of low doses of auxin transport inhibitors to the stem of SL mutants of *Arabidopsis* led to a phenotype close to that of wild-type plants, in accordance with a main role of auxin transport in determining the number of buds that grow out into branches (Bennett et al., 2006; Lazar and Goodman, 2006; Lin et al., 2009). However, even if several biological and modeling pieces of evidence support the canalization theory, the nature of the mechanism inducing export of axillary bud auxin into the stem is still relatively abstract (Prusinkiewicz et al., 2009).

### Dynamic Regulation of Bud Outgrowth Along a Same Axis

The release of apical dominance leads to bud outgrowth at given positions on the plant depending on the plant species. Outgrowth of these buds then inhibits outgrowth of the other buds on the axis (Morris, 1977). In garden pea, the inhibition exerted by a growing bud on the buds below was related to auxin synthesized and exported by the growing bud and transported downward in the main stem (Balla et al., 2016). This mechanism limits excessive branching that may be detrimental for the plant.

SLs also appear as main components of this phenomenon and could act through a double feedback process (Dun et al., 2009b). In a first feedback, branching initiation increases SL biosynthesis through a branch-derived signal, probably auxin, which could contribute to further inhibit bud outgrowth. This regulation scheme was identified from the experimental observation that the initiation of a new branch in garden pea correlated locally with the up-regulation of SL biosynthesis genes in the corresponding node, and this upregulation was prevented by branch removal (Dun et al., 2009b). Second, SL deficiency in the node, which contributes to promote bud outgrowth, activates a feedback signal that up-regulates SL biosynthesis and decreases CKs in the xylem sap, thus contributing to prevent bud outgrowth. At the origin of this hypothesis, SL mutants of different species (except pea *rms2*) were observed displaying reduced CKs in the xylem sap and higher expression of SL biosynthesis genes, while exogenous SL supply repressed SL biosynthesis (Foo et al., 2005; Snowden et al., 2005; Foo et al., 2007; Drummond et al., 2009; Hayward et al., 2009). Computer simulations support this double SL-based regulating system in pea branching regulation as they capture the overall experimental phenotypes of branching, SL biosynthesis gene expression, and xylem-sap CKs that are observed for different graft combinations between mutant and wild-type pea (Dun et al., 2009b).

In garden pea, the feedback signal derived from SL perception is dependent on *RMS2* and moves from shoots to roots (Beveridge, 2000; Foo et al., 2005; Foo et al., 2007). The chemical nature of the *RMS2*-dependent feedback has been extensively discussed (Ongaro and Leyser, 2008; Dun et al., 2009a). Ligerot et al. (2017) recently demonstrated that protein RMS2 functions as an auxin receptor. They also observed that SL root-feeding, as a disruption of auxin transport, repressed auxin biosynthesis in the shoot. This suggests the existence of a feedback loop in which auxin depletion in the stem stimulates SL biosynthesis in an RMS2-dependent manner in the roots, which in turn stimulates auxin biosynthesis in the shoot.

### Contribution of Roots to Bud Outgrowth

As mentioned above, CK and SL biosynthesis in the shoot are main components of auxin-mediated apical dominance. But CKs and SLs are also synthesized in roots and root-derived CKs and SLs are transported in the shoots through the xylem and also contribute to stimulate and inhibit shoot branching, respectively (Beveridge, 2000; Young et al., 2014; Muller et al., 2015).

Root-derived CKs were long believed to contribute to the bud outgrowth response to decapitation because the xylem-sap CK content increases after decapitation and accumulates in buds, and this is prevented by exogenous auxin supply (Bangerth, 1994; Turnbull et al., 1997; Mader et al., 2003). However, the absence of a rapid response of CK-related biosynthesis genes in roots indicates that root-derived CKs may have a secondary role in this process (Tanaka et al., 2006). Recent experiments comparing root-bearing plants and root-depleted isolated nodal stem segments indicate that root-derived CKs may in fact antagonize the effect of auxin in apical dominance. Decapitated plants of garden pea SL mutants were indeed unresponsive to auxin supply, due to the antagonistic effect of root-derived CKs on the inhibitory effect of auxin, while the isolated nodal stem segments (without root-derived CKs) were auxin responsive (Young et al., 2014). In *Arabidopsis*, intact auxin-producing CK-synthesis/signaling mutants were accordingly less branched than wild-type plants, while the isolated nodal segment bud response to auxin was not impaired in CK mutants as compared to the wild-type (Muller et al., 2015). Since CK biosynthesis in the roots is promoted by high nitrogen nutrition (Takei et al., 2002; Xu et al., 2015), root-derived CKs could antagonize auxin-mediated apical dominance in case of a high soil nitrogen content by modulating the shoot CK levels. In line with this, CK mutants of *Arabidopsis* exhibited an altered positive branching response to an increase in the soil nitrogen conditions (Muller et al., 2015). On the opposite, root-derived SLs, sensitive to phosphate or nitrogen deficiency or water stress (Ha et al., 2014; Cochetel et al., 2018; Mostofa et al., 2018), could strengthen auxin-mediated apical dominance in case of a low soil nutrient status or water stress. Accordingly, root-derived SLs have been reported to mediate the effect of soil phosphate deficiency on shoot branching (Kohlen et al., 2011; Xi et al., 2015).

### Regulation of Bud Outgrowth by Other Hormones

Abscisic acid (ABA) is well known for its role in plant adaptation to abiotic stresses (Vishwakarma et al., 2017), and gibberellins (GAs) modulate a range of processes such as cell elongation and fruit maturation (see Olszewski et al., 2002; Yamaguchi, 2008; Hartmann et al., 2011; Ragni et al., 2011). They both take part to bud outgrowth regulation, but their role has been less investigated than the roles of auxin, CKs, and SLs.

The effect of GAs on bud outgrowth varies strongly among species. GAs inhibit shoot branching in rice (Lo et al., 2008; Ito et al., 2018), bahiagrass (Agharkar et al., 2007), *Arabidopsis* (Silverstone et al., 1997), hybrid aspen (Mauriat et al., 2011), and tomato (Martinez-Bello et al., 2015). The exact mechanism behind their effect remains elusive and might be linked to the modification of SL biosynthesis (Ito et al., 2017) and an increase of sugar sink strength (see below) (Buskila et al., 2016). In perennial woody plants such as rose and *Jatropha curcas*, GAs are promoters of bud outgrowth (Choubane et al., 2012; Ni et al., 2017). In apple, exogenous application of GAs to axillary buds did not promote outgrowth (Tan et al., 2018).

The role of ABA as an inhibitor of bud outgrowth was long hypothesized based on the observations that exogenous ABA supply inhibits bud outgrowth (White and Mansfield, 1977; Chatfield et al., 2000; Cline and Oh, 2006; Corot et al., 2017; Yuan et al., 2018) and that the bud ABA content is negatively correlated to the bud ability to grow out. In particular, the bud ABA level decreases in response to decapitation and increases in response to exogenous auxin supply in annual plants (Eliasson, 1975; Everatbourbouloux and Charnay, 1982; Knox and Wareing, 1984; Gocal et al., 1991), and ABA accumulates during cold-induced bud dormancy in perennial plants (Rohde et al., 1999; Arora et al., 2003; Wang et al., 2016a). Mutants recently confirmed a role of ABA in bud outgrowth regulation. *Arabidopsis* mutants deficient in ABA biosynthesis (*nced3-2* and *aba2-1*) displayed higher bud outgrowth frequency (Reddy et al., 2013; Yao and Finlayson, 2015). Similarly, genetically altered poplar with reduced sensitivity to ABA exhibited enhanced shoot branching (Arend et al., 2009).

ABA has been reported to act downstream of auxin signaling (AUXIN-RESISTANT 1 *AXR1*), MORE AXILLARY BRANCHED (MAX) signaling (*MAX2*), and BRANCHED1 (*BRC1*) gene (Gonzalez-Grandio et al., 2013; Yao and Finlayson, 2015; Gonzalez-Grandio et al., 2017). *AtBRC1* directly induces ABA synthesis in the bud by upregulating the expression of *9-CIS-EPOXICAROTENOID DIOXIGENASE 3* (*NCED3*), which encodes a key ABA-synthesis enzyme (Yao and Finlayson, 2015; Gonzalez-Grandio et al., 2017). ABA may partly inhibit bud outgrowth by reducing auxin biosynthesis and transport within the bud and also cell multiplication (Yao and Finlayson, 2015), which may impair bud capacity to export its own auxin and to grow out (Prusinkiewicz et al., 2009). ABA is also synthesized outside the bud and can access the buds (Everatbourbouloux, 1982; Lacombe and Achard, 2016). This raises the question of the role of such externally synthesized ABA in the control of bud outgrowth. ABA exogenously supplied to the stem below the bud inhibited bud outgrowth but did not do so when supplied above the bud, indicating a likely preferential role of upstream xylem-transported ABA (Cline and Oh, 2006). In barley, ABA was reported to suppress SL biosynthesis in the basal part of the plant and roots, which in this case promoted tiller emergence (Wang et al., 2018). These findings indicate the complexity of ABA-dependent bud outgrowth regulation and its interactions with other branching-related hormonal networks.

### Summary

**Figure 1:**
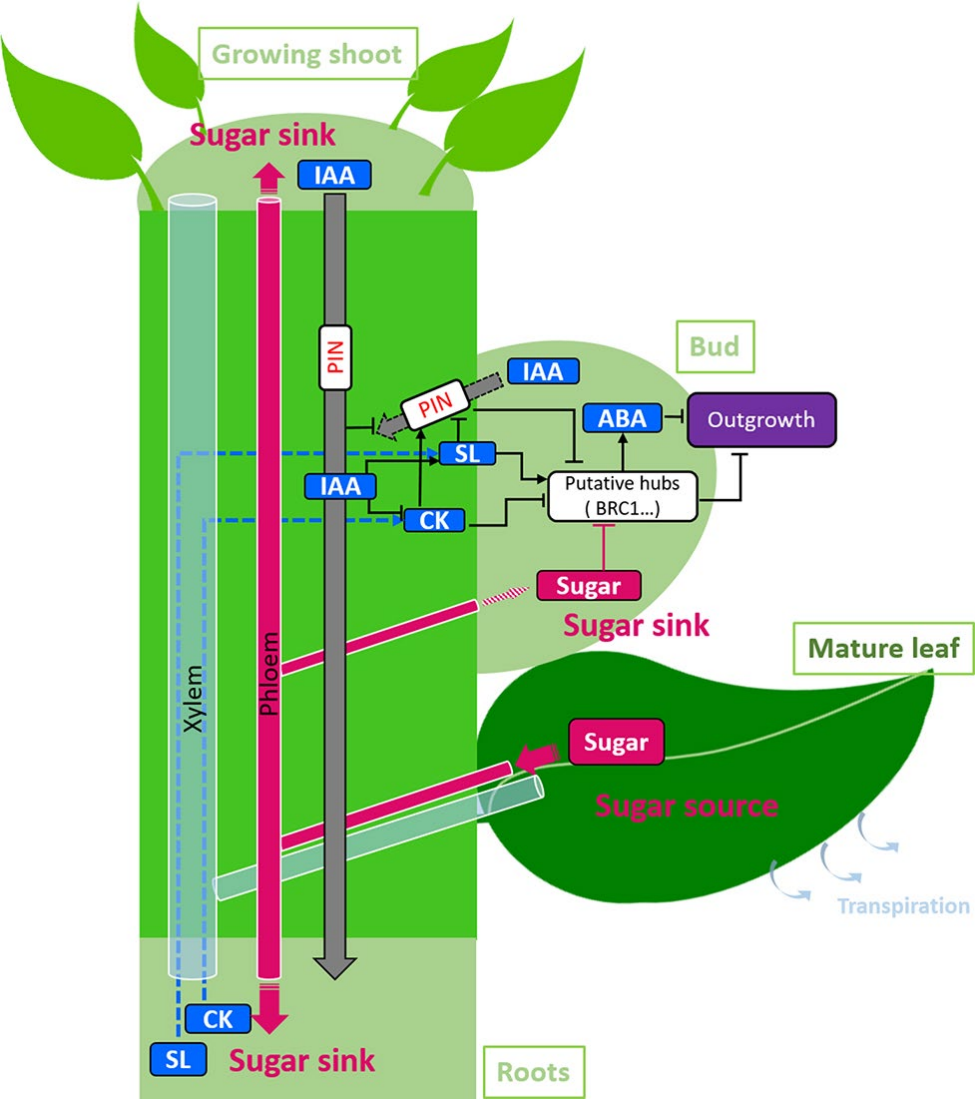
Regulation of the outgrowth of one bud by sugars and hormones on a growing shoot. Sugars are produced by photosynthetic organs and transported by mass flow in the phloem to sugar sinks, *i.e.*, the growing organs (apical leaves and internodes, elongating branches, roots). Auxin (IAA) is produced by apical growing organs and is transported in the stem to the roots through PIN proteins (gray arrow). Auxin in the nodal stem down- and up-regulates the biosynthesis of cytokinins (CKs) and strigolactones (SLs), respectively and prevents auxin export out of the bud through the canalization process. Sugars, CKs, SLs, and auxin export are integrated in the bud by hubs, which include the gene *BRC1*, to control bud outgrowth. In addition, CK and SL syntheses in the roots and their transport upwards in the xylem by the transpiration stream may increase the CK and SL contents in the nodal stem (dotted arrows). ABA acts downstream of *BRC1* to inhibit bud outgrowth. The feedback loops between hormones are not represented. The hormonal regulators are represented in blue, and sugars in pink. Sugar sinks are represented in light green, sugar sources in dark green. Black arrows represent the effects of one regulator on a target. Large arrows represent active transport processes. The way sugars are transported to the bud is unknown and is represented by a large dashed arrow.

So far, many studies have focused on understanding how auxin, synthesized by growing apical organs and transported downwards through the stem in annual plants, acts to inhibit the outgrowth of a bud without entering the bud during apical dominance. They highlighted an intricate regulatory network described in **Figure 1** that displays two pathways. In the first pathway, auxin acts through a canalization mechanism that creates a main flux of auxin downwards and inhibits the initiation of auxin fluxes from lateral buds. In the second pathway, auxin acts more locally through its concentration in the node which modulates CK and SL biosynthesis, which in turn relay the auxin signal from the stem to the bud. CK and SL signals are integrated in the bud through BRC1-dependent and -independent pathways. *BRC1* acts at least partly by up-regulating ABA biosynthesis.

The relationship between both pathways is not fully understood. Both pathways probably interact because SLs also control auxin transport, and CKs were recently reported to control auxin efflux carrier proteins (PIN3, PIN4, PIN7) in *Arabidopsis* (Waldie and Leyser, 2018). From a temporal point of view, auxin transport regulation could come after local regulation, as indicated experimentally for garden pea (Chabikwa et al., 2019). Intriguingly, the role of CKs as a second messenger has recently been questioned for *Arabidopsis* because isolated nodal segments of mutants deficient in CK biosynthesis or signaling exhibited a normal response to exogenous auxin (Muller et al., 2015).

Besides these well-studied auxin-dependent pathways in the vicinity of the bud, some studies have also highlighted the important role of roots as a source of SLs and CKs. They indicate that auxin-related apical dominance in the shoot may be modulated by root-derived CKs and SLs, as a way to adjust apical dominance in the aerial part to soil nutrient status (Kohlen et al., 2011; Muller et al., 2015; Xi et al., 2015). Furthermore, evidences support a main role of root-derived CKs and SLs in regulating the outgrowth pattern along an axis through feedback loops between shoot and root, and SLs, CKs, and auxin (not represented on the figure; Dun et al., 2009b; Ligerot et al., 2017). However, research about the role of long-distant components from buds other than auxin is still scarce, and further work is definitely required to get a more integrated understanding of the hormonal regulation of apical dominance expression in plants.

## Bud Outgrowth Regulation Through Competition for Nutrients

Relationships between the plant nutrient status and the number of new branches arising in a growth period have been suggested for decades. For example, for tree species, the number of new branches was found correlated to the vigor of the parent branch (Heuret et al., 2000). In wheat, the bud outgrowth probability at a given leaf rank on the main stem was correlated to the parent leaf mass *per* unit area (Bos, 1999; Evers et al., 2006). In ryegrass, the number of tillers that recovered after cutting was strongly correlated to the initial carbohydrate level before cutting (Davies, 1965). These observations led to the intuition that the degree of competition for nutrients within the plant regulates the investment into new branches.

The degree of competition within the plant is a complex variable that depends on nutrient supply to the plant, nutrient transport, storage, and use by the different organs and evolves dynamically with plant development. To address this complexity, different hypotheses related to competition for nutrients in shoot branching were first tested using computational models. The results of these simulations confirmed the plausibility of branching regulation by nutrient competition. These results have been corroborated in recent years by physiological experiments that brought evidence supporting the initial intuition and providing a better understanding of the mechanisms involved.

### Computer Models of Branching Regulation by Competition for Nutrients

The first models were developed for trees and formalized that a limited amount of nutrients is assimilated by the plant and shared among tree branches according to given priority rules, and that the nutrient level in a branch determines the emergence of new branches. Based on the observations of weaker water flow in less vigorous branches as compared to the main trunk (Zimmermann, 1978), 25 years ago, Borchert and Honda implemented a model in which branches were in competition for nutrients coming from the roots through the transpiration stream (Borchert and Honda, 1984). Later, the model LIGNUM initially developed for young pine trees considered that branches were in competition for carbohydrates produced by photosynthesis in tree aerial parts (Perttunen et al., 1996; Perttunen et al., 1998). Competition for nutrients or carbohydrates among branches simulated qualitative observations made on real trees, such as a reduction of branch emergence with tree development or branching stimulation after branch removal by pruning (Borchert and Honda, 1984). Priority rules for nutrient or carbohydrate allocation among branches were essential to simulate observed tree forms (Perttunen et al., 1998; Palubicki et al., 2009). For example, in Borcher and Honda’s model, preferential nutrient allocation to a given branch position and to the more vigorous branch (defined by the number of daughter transpirating branches) explained the morphological differentiation of branches into leaders and weaker lateral shoots observed in some species (Borchert and Honda, 1984).

Several years later, the concept emerged that competition for carbohydrates can be represented by the source-sink ratio (Warren-Wilson, 1972; Lacointe, 2000), which is the balance between the production rate of carbohydrates by photosynthesis and their utilization rate for growth. For grass species, this concept arises in particular from the observed correlations between the tillering level and the balance between (i) PAR intensity, that determines photosynthesis, and (ii) temperature, that determines the organ growth rate (Mitchell, 1953; Bos and Neuteboom, 1998). Based on findings that sugars act as signaling entities on meristematic activity (Sherson et al., 2003; Heyer et al., 2004), the authors of the rice model ecomeristem assumed that sugars also acted as signals in bud outgrowth regulation and that the source-sink ratio was a signal analogous to sugar signaling (Luquet et al., 2006). Supporting the concept, the source-sink ratio dynamics correlated with the dynamics of sugar reserves, an indicator of sugar availability. The authors argued that such a regulating system allowed for the plant to adjust the carbohydrate sinks to the sources: in case of a high source-sink ratio, plant development is stimulated, thus increasing the sink strength for carbohydrates, which in turn decreases the ratio. This concept has been taken up by other models, *e.g.,* for wheat (Evers et al., 2010) or trees (Letort et al., 2008; Mathieu et al., 2009). Simulations of plant development were validated against quantitative experimental observations for grasses, but the robustness of the models was not demonstrated (Luquet et al., 2006; Evers et al., 2010). For trees, the concept explained observed trends qualitatively, such as a low branch number under low light intensity, or branching rhythmicity as a result of the negative feedback between branch emergence and the source-sink ratio (Letort et al., 2008; Mathieu et al., 2009).

All these studies show that branching regulation by competition for nutrients explains some of the observed plant behaviors in different species. However, the concept was lacking more direct molecular experimental evidence. In the 2010s, several biological experiments, independent of modeling studies, confirmed bud outgrowth regulation by competition for carbohydrates and the involvement of sugar signaling in some species.

### Experimental Evidence for Bud Outgrowth Regulation by Competition for Carbohydrates in Grasses and Garden Pea

A first series of experiments demonstrated that differences in tiller bud outgrowth induced by changes in the source-sink ratio in some grass species were correlated to differences in the bud sugar status. The *tin* mutant of wheat, which is characterized by earlier internode elongation as compared to the wild type, displayed a reduced number of tillers and over-expression of a sucrose-starvation gene, downregulation of a sucrose-inducible gene, and a reduced sucrose content in the inhibited buds (Kebrom et al., 2012). In sorghum, bud outgrowth inhibition by defoliation was correlated to up- and down-regulations of sucrose starvation and sucrose-inducible genes in buds, respectively (Kebrom and Mullet, 2015). In this case, defoliation of the subtending leaf blade or any other leaf blade inhibited bud outgrowth, indicating that outgrowth may be dependent on the overall plant sugar status, as implemented in models, rather than on sugar supply by the subtending leaf. No similar studies were made in tree species.

Definitive proof of a role of sugars in bud outgrowth regulation by the source/sink balance was given by Mason et al. (2014) in garden pea. Removal of the apical growing organs by decapitation of the shoot tip led to bud outgrowth and rapid sugar redistribution and accumulation in the outgrowing buds before auxin depletion in the nodal segment adjacent to the outgrowing bud. This phenomenon was abolished by defoliation which reduced sugar supply, while exogenous sucrose supply through the petioles of intact plants (not decapitated or defoliated) released the buds from apical dominance. These behaviors indicate that sugar accumulation in the buds of decapitated plants is both necessary and sufficient for bud outgrowth. Additional proof was given recently by the observation that the elevated sucrose and hexose levels of transgenic plants overexpressing fructose 1,6-bisphosphatase II in the cytosol increased the number of lateral shoots (Otori et al., 2017).

### Role of Sugar in Bud Outgrowth Regulation

Using excised nodal stem segments *in vitro* to manipulate sugar availability for buds easily, evidence was brought about both the trophic and signaling roles of sugars in bud outgrowth, as demonstrated in other processes of plant development (Moore et al., 2003; Rolland et al., 2006; Lastdrager et al., 2014; Li and Sheen, 2016; Sakr et al., 2018). As compared to an osmotic control, sucrose supply or supply of its derivative hexoses (glucose and fructose) to isolated buds increased sugar levels in buds and stimulated their outgrowth in a dose-dependent manner in species such as rose and garden pea (Henry et al., 2011; Rabot et al., 2012; Barbier et al., 2015; Fichtner et al., 2017). In line with the trophic role of sugars, sugar-induced bud outgrowth in rose was characterized by a higher sugar metabolic activity of the bud linked to increased expression of the sugar transporter *RhSUC2* and in the expression and activity of vacuolar invertase *RhINV1*, an enzyme responsible for sucrose cleavage into hexoses and usually related to organ sink strength (Girault et al., 2010; Henry et al., 2011; Rabot et al., 2012). Interestingly, non-metabolizable sucrose or fructose analogs also induced bud outgrowth in rose (Rabot et al., 2012; Barbier et al., 2015; Wingler, 2018) and stimulated the expression and the activity of the vacuolar invertase *RhINV1* (Rabot et al., 2012). This observation supports a scenario in which sugar availability for the bud acts as a signaling entity regulating its outgrowth and its sink strength. This role may be mediated, at least partly, through trehalose-6-phosphate, an important indicator of the carbohydrate status in plants (Figueroa and Lunn, 2016). Sucrose supply to nodal stem segments of garden pea induced a rapid concentration-dependent increase of the trehalose-6-phosphate (Tre6P) content in the buds that was highly correlated with their outgrowth rate (Fichtner et al., 2017). Such a rapid Tre6P increase in outgrowing buds was also observed after removal of the main sink for sugars by decapitation of garden pea shoots. Sugar signal may regulate bud outgrowth through the sucrose non-fermenting kinase 1 (SnRK1) complex, which perceives cell energetic status and regulates growth activity accordingly (Tsai and Gazzarrini, 2014). This supports the concept implemented in models that the source-sink ratio controls a sugar signal that modulates bud outgrowth.

### Sugar Interplays With Hormones

Interplays between sugar and hormonal pathways have been recently reported in bud outgrowth regulation in rose and pea (Barbier et al., 2015; Bertheloot et al., 2019). Bud outgrowth is under an antagonistic coupled control of sugar and auxin levels. While exogenous auxin supply to nodal segments *in vitro* inhibited bud outgrowth dose-dependently, sugar supply partially removed the inhibitory effect of auxin in a manner that was also dose-dependent. This supports the view that a high plant sugar status may attenuate auxin-mediated apical dominance, leading eventually to bushy phenotypes.

Sugar promoting effect on bud outgrowth was accompanied by a number of changes in the bud outgrowth hormonal network for rose nodal segments *in vitro* (Barbier et al., 2015). These changes include the simulation of CK biosynthesis and level in the stem and a down-regulation of a SL signaling gene (*MAX2*). However, CK level in the stem and auxin export from the bud to the stem are unlikely to be the main mediators of sugar promoting effect on bud outgrowth. Without sucrose, CK supply to rose nodal segments *in vitro* did not induce bud outgrowth, and sucrose could not antagonize the auxin-dependent repression of CK levels in the stem (Barbier et al., 2015; Bertheloot et al., 2019). Sugar-stimulated bud outgrowth was rather related to the impairment of SL response, because exogenously applied SL was inefficient in inhibiting bud outgrowth in the presence of high sugar concentration in rose and pea (Bertheloot et al., 2019). In addition, buds of pea mutants deficient in SL perception displayed a reduced response to changes in sugar supply *in vitro*. Finally, a computational model, in which auxin regulates bud outgrowth through regulation of the production of CKs and SLs (second messenger model) and sugar acts by suppressing SL response, captured the diversity of observed bud outgrowth responses to sugar and hormones in a quantitative manner. Further studies are required to decipher the exact targets of sugars, but the SL signaling-related gene *MAX2* and the integrator gene *BRC1* that are downstream of SLs and down-regulated under high sucrose conditions for different species may be involved (Kebrom et al., 2010; Kebrom et al., 2012; Mason et al., 2014; Barbier et al., 2015; Kebrom and Mullet, 2015; Otori et al., 2017).

### Summary

These data highlight that competition for sugars within the plant, indicated by the source/sink ratio, is a key component of branching regulation at least in annual species. As depicted in **Figure 1**, sugars are produced by source organs, mainly photosynthetically leaves, and transported through the phloem to sink organs such as the shoot growing apical and root zones. High sugar availability in the vicinity of the bud, resulting from high ratio of source to sink activity, promotes bud outgrowth. The exact pathway by which sugar availability regulates bud outgrowth remains to be elucidated, but sugar signaling seems crucial (Rabot et al., 2012; Barbier et al., 2015; Fichtner et al., 2017). Such signaling role of sugar appears as an efficient way to adjust plant development to endogenous resources. New branches, which are highly demanding in resources, are created only if the resource status of the plant is sufficient to sustain their growth.

Recent studies highlight the existence of an interplay between sugar and the hormonal networks in bud outgrowth regulation; more particularly, high sugar availability antagonizes auxin inhibitory effect through inhibition of SL signaling (Bertheloot et al., 2019). A hormonal role has also been suggested by the simulations of previous nutrient-based models. Indeed, this kind of models could not fully explain branching phenotypes at the plant scale and should be coupled to other signaling processes. We report that Borchert and Honda’s and LIGNUM models include priority rules for nutrient allocation among branches, essential to simulate tree branching habits (Borchert and Honda, 1984; Perttunen et al., 1996; Perttunen et al., 1998). Other models have to define which bud is sensitive to carbohydrates to simulate positions of branches on trees (Letort et al., 2008) or the observed coordination between tiller appearance and parent axis development in grasses (Letort et al., 2008; Evers et al., 2010). Sensitivity to carbohydrates also depends on mineral nutrition in grasses (Dingkuhn et al., 2007; Kim et al., 2010a; Kim et al., 2010b; Alam et al., 2014). All these effects may involve hormonal pathways, because hormones are regulated by both plant development and growth conditions. This raises the question of how sugar and hormonal signals are integrated to regulate bud outgrowth in spatial and temporal dynamics at the plant scale.

Contrary to sugars, the role of xylem-transported nutrients in bud outgrowth regulation has been the subject of very few studies. However, they could contribute to bud outgrowth regulation. Amino acids were required for bud outgrowth in nodal segments of rose *in vitro* (Le Moigne et al., 2018), transgenic lines deficient in amino acids displayed decreased tillering in rice (Funayama et al., 2013; Ohashi et al., 2017; Ohashi et al., 2018), and overexpression of a glutamine synthase gene promoted tillering in sorghum (Urriola and Rathore, 2015). Whether amino acids act as signaling entities in bud outgrowth remains to be investigated.

## Interaction of Light With the Network of Endogenous Regulators

Besides its role as an energy source for photosynthesis, light is also a powerful environmental signal that controls many developmental processes (de Wit et al., 2016; Gangappa and Botto, 2016; Lee et al., 2017). In particular, it is involved in the shade avoidance syndrome (SAS), characterized by typical morphological changes such as leaf hyponasty, an increase in hypocotyl and internode elongation, and extended petioles, which aim to maximize light interception by the plant for photosynthesis (Franklin, 2008). In bud outgrowth regulation, light also acts as a signal that may prevent a new branch from developing in low light conditions. In accordance with the signaling role of light, a very low light intensity on the bud was sufficient to trigger bud outgrowth in decapitated rose (Girault et al., 2008). Tillering can cease in grasses before the occurrence of a significant reduction in PAR intensity due to canopy closure, but concomitantly with a reduction of the R:FR ratio (Ballare et al., 1987). Simulation studies support a role of light in shaping plant branching architecture in different species. In trees, the global branching structure can be explained qualitatively by space colonization algorithms, which consider competition for space as the key factor determining the branching structure of the tree (Runions et al., 2007; Palubicki et al., 2009). In herbaceous species, the inhibiting effect of shading or high plant densities can be simulated by regulating bud outgrowth by the local light environment on the apical meristem at the time of bud formation (Gautier et al., 1999; Evers et al., 2007).

At the plant scale, light signaling interacts with hormonal and/or nutrient regulation by controlling the homeostasis, transport, and signaling of hormones and nutrients. Remarkably, light, hormones, and nutrients seem to converge to the same regulating hubs (Quail, 2002; Moore et al., 2003; Lau and Deng, 2012; Li et al., 2017; Mawphlang and Kharshiing, 2017; Sakuraba and Yanagisawa, 2018; Simon et al., 2018). Compared to the endogenous network responsible for apical dominance, relatively few studies have focused on the interaction of light with hormones and nutrients in the control of axillary bud outgrowth. Most studies have focused on the effect of the R:FR ratio, which is a signal of canopy closure. More recently, the effect of light intensity was also investigated.

### Interaction of Light With the Hormonal Regulatory Network

#### R:FR Ratio

Studies were made by directly manipulating light quality or by using *phyB Arabidopsis* mutants, which are deficient in phytochrome B-mediated red light perception and display a low branching level as compared to the wild-type (Kebrom et al., 2006; Finlayson et al., 2010; Su et al., 2011). Those studies highlight that enhanced ABA biosynthesis in the bud has a main role in the effect of the R:FR ratio on bud outgrowth. The bud outgrowth response to R:FR is negatively correlated to the bud ABA level and to the expression of ABA biosynthesis- and signaling-related genes in different species (Tucker and Mansfield, 1972; Gonzalez-Grandio et al., 2013; Reddy et al., 2013; Yao and Finlayson, 2015; Kebrom and Mullet, 2016; Gonzalez-Grandio et al., 2017; Holalu and Finlayson, 2017; Tarancon et al., 2017; Yuan et al., 2018). The ABA response was even reported to precede the bud outgrowth response to an increase of the R:FR ratio in *Arabidopsis* (Holalu and Finlayson, 2017). Furthermore, *Arabidopsis* mutants deficient in ABA biosynthesis (*nced3-2* and *aba2-1*) exhibited lower suppression of bud outgrowth by low R:FR than the wild type (Reddy et al., 2013; Yao and Finlayson, 2015). The mechanisms leading to changes in the ABA level involve *BRC1*. *BRC1* induces ABA biosynthesis in buds, is up-regulated by low R:FR or following *phyB* mutation, and is involved in low R:FR-dependent branch suppression (Gonzalez-Grandio et al., 2013; Yuan et al., 2018). Low R:FR-induced ABA biosynthesis may repress bud outgrowth partly by reducing bud auxin biosynthesis, since both *phyB Arabidopsis* mutants and exogenous ABA supply to wild-type plants reduced the expression of an auxin biosynthesis gene within the bud (Finlayson et al., 2010; Yao and Finlayson, 2015).

Upstream of *BRC1*, several other regulators of bud outgrowth than ABA could contribute to bud inhibition by low R:FR. Auxin plays a key role in the shade-avoidance syndrome, including the promotion of hypocotyl and petiole growth, leaf hyponasty, and phototropism (Iglesias et al., 2018). In seedlings, low R:FR increases auxin level in the foliage by stimulating its biosynthesis; auxin then moves to the stem where it reaches epidermal tissues through lateral orientation of PIN proteins to drive the auxin flux to the epidermis to promote growth (Iglesias et al., 2018). Similarly, relationships have been observed between auxin and bud outgrowth inhibition in *Arabidopsis phyB* mutants, which cannot perceive red light. The branching inhibition reported in *phyB Arabidopsis* mutants was alleviated by disrupting auxin signaling (Finlayson et al., 2010). In this case, branching inhibition was related to elevated auxin sensitivity and signaling in the shoot segments proximal to axillary buds (Reddy and Finlayson, 2014). Low auxin level supply to isolated stem segments inhibited *phyB* buds more than wild-type, and *phyB* shoots displayed elevated auxin-responsive genes expression compared to the wild-type. This obviously raises the question of how auxin- and ABA-mediated pathways interact to regulate bud outgrowth in response to R:FR. Although ABA acts downstream of auxin signaling (Yao and Finlayson, 2015), Holalu and Finlayson (2017) reported that bud response to low R:FR involve changes in bud ABA signaling before any detectable alteration in stem auxin signaling, indicating that ABA and auxin signalings are part of different R:FR-induced pathways. ABA pathway may be responsible for a rapid response of the bud to R:FR, while auxin signaling in the stem may sustain this rapid response. Low auxin transport rate was also observed in the shoots of *phyB* mutants but its role in inhibiting bud outgrowth was not demonstrated (Reddy and Finlayson, 2014).

Besides auxin, SL biosynthesis- and signaling-related genes were also found to be up-regulated by low R:FR or by *phyB* mutation in chrysanthemum, sorghum, or petunia buds (Kebrom et al., 2010; Drummond et al., 2015; Yuan et al., 2018). Furthermore, bud outgrowth inhibition by *phyB* mutation was impaired in SL biosynthesis (*max4*) or signaling (*max2*) mutants as compared to wild-type *Arabidopsis* (Finlayson et al., 2010), indicating a potential role of these genes in low R:FR-dependent bud outgrowth regulation. This is in accordance with the main role of the SL signaling-related gene *MAX2* in light-regulated hypocotyl elongation in *Arabidopsis* seedlings (Shen et al., 2007; Shen et al., 2012; Jia et al., 2014). Future tasks would be to identify the role of *MAX2* and understand its relationship with ABA and auxin signaling pathways in bud response to R:FR.

#### Light Intensity

The interaction between light intensity and hormonal regulation of bud outgrowth has mainly been investigated in rose. First data indicate that GAs are not sufficient to mimic the promotive effect of light in dark-placed buds (Choubane et al., 2012). For decapitated plants, dark-repressed bud outgrowth correlated with a down-regulation of two GA biosynthesis genes, and light-induced bud outgrowth was inhibited by GA biosynthesis inhibitors, but GA supply to plants in the dark could not rescue bud outgrowth.

Recent experimental studies on rose support a model in which light intensity stimulates CK biosynthesis in the stem, which in turn stimulates bud outgrowth. As compared to darkness or low light intensity, a higher light intensity rapidly and significantly increased the CK content in the nodal segment bearing the light-stimulated bud (Roman et al., 2016; Corot et al., 2017). This was correlated with rapid up-regulation of genes encoding CK synthesis, transport and signaling, and down-regulation of genes encoding CK degradation (*RhCKX1*) (Roman et al., 2016). This is in line with the known effect of light on CK biosynthesis, metabolism, and transport in other biological processes (Zubo et al., 2008; Boonman et al., 2009; Zdarska et al., 2015; Janeckova et al., 2018). In addition, local exogenous CK application restored the bud outgrowth ability under non-permissive light conditions (Roman et al., 2016; Corot et al., 2017). Interestingly, studies on the shoot apical meristem in tomato and *Arabidopsis* also demonstrated the involvement of CKs in the light-induced activity of the apical meristem (Yoshida et al., 2011; Pfeiffer et al., 2016).

Light-induced bud outgrowth may involve the two CK-related processes controlling bud outgrowth: *BRC1* repression and PIN up-regulation (which would increase auxin canalization capacity) (Dun et al., 2012; Waldie and Leyser, 2018). Indeed, both light and CK exogenous supply down-regulated *BRC1* in the bud and up-regulated *PIN1* expression in the stem for rose decapitated plants (Roman et al., 2016). In line with this, light intensity was also reported to down-regulate *BRC1* in *Arabidopsis* (Su et al., 2011). In addition, both light and CKs supply to rose decapitated plants decreased the expression of the SL signaling-related gene *MAX2* and up-regulated sugar metabolism-related genes (Djennane et al., 2014; Roman et al., 2016), consistent with the well-known role of CKs on the strength of sink organs (Roitsch and Ehness, 2000; Wang et al., 2016b). For rose intact plants, high light intensity also decreased ABA level in the node adjacent to the bud compared to low light intensity, and ABA exogenous supply to the node could antagonize the promoting effect of CK supply under low light intensity (Corot et al., 2017). All these changes underline the complexity of the regulation, and further research is required to understand the basic mechanism behind the light effect on bud outgrowth.

Besides CKs located in bud vicinity, it is likely that root-derived CKs contribute to bud outgrowth stimulation in response to light intensity. Indeed, the concentration in root-derived CK forms (tZ, tZR, tZRMP) increases in stems and buds in these conditions (Roman et al., 2016; Corot et al., 2017); however, this remains to be demonstrated experimentally.

### Interaction of Light With the Nutrient-Based Regulatory Network

Strong evidence is given about a main role of competition for carbohydrates, indicated by the source-sink ratio, in bud outgrowth regulation in garden pea and grasses (Kebrom et al., 2012; Kebrom and Mullet, 2015). The carbohydrate source-sink ratio may be affected by the plant light environment: a low R:FR ratio enhances stem growth (Demotes-Mainard et al., 2016), a strong sugar sink, and PAR intensity regulates photosynthesis as well as plant aerial morphogenesis and root growth (Granier and Tardieu, 1999; Chenu et al., 2005; Nagel et al., 2006). As proposed in some tillering models (Luquet et al., 2006; Evers et al., 2010) and by Kebrom (2017) and Bertheloot et al. (2019), light regulation of source-sink relationships within the plant may modulate sugar availability for buds, leading in turn to reduced auxin-related apical dominance and induction of bud outgrowth. This is supported by studies reporting a negative impact of a low R:FR ratio on the sugar content or on genes related to sugar metabolism and signaling in the bud (Kebrom and Mullet, 2016; Yuan et al., 2018), as well as changes in stem sugar levels in response to light intensity, in ways correlated to bud outgrowth (Lafarge et al., 2010; Furet et al., 2014; Corot et al., 2017). However, the involvement of sugar in the effect of light has not been proved by physiological experiments yet.

Experimental data rather indicate that local sugar availability in the stem or in the bud may not be limiting for bud outgrowth in case of low PAR intensity. In decapitated and defoliated rose plants under white light, preventing light perception by the bud by masking it while leaving the photosynthetic stem under white light maintained the bud inhibited, while applying a photosynthesis inhibitor on the bud did not prevent its outgrowth (Girault et al., 2008; Roman et al., 2016). In addition, local exogenous sugar supply to decapitated shoot stumps under darkness, to the petioles of intact plants under low PAR intensity, or to rose nodal segments cultivated *in vitro* in darkness did not induce bud outgrowth (Henry et al., 2011; Rabot et al., 2012; Roman et al., 2016; Corot et al., 2017). The activity of isolated apical meristems of *Arabidopsis* was also prevented by darkness and was not restored by exogenous sugar supply (Yoshida et al., 2011; Pfeiffer et al., 2016; Li et al., 2017). For both apical meristem and axillary buds under limiting light conditions, CKs may be a limiting factor explaining the inability of sugars to promote bud outgrowth locally. CKs could act by limiting the bud sink strength for sugars (Albacete et al., 2014).

### Summary

**Figure 2:**
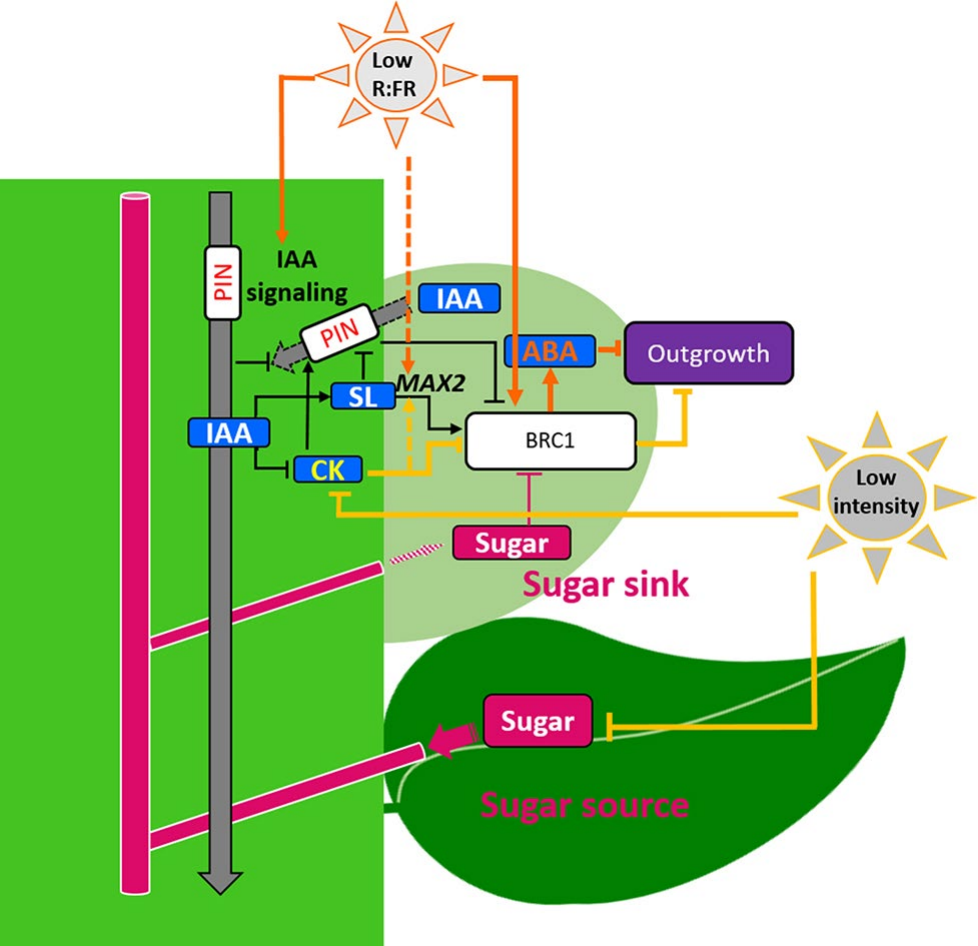
Interaction of light intensity and the R:FR ratio with the endogenous regulators of bud outgrowth. A low R:FR ratio stimulates ABA production in the bud, which inhibits bud outgrowth, a phenomenon that is reinforced later on by auxin signaling stimulation through an unknown mechanism (solid dark orange arrows and text); low R:FR also up-regulates the SL signaling-related gene *MAX2* (dotted dark orange arrows), but the contribution of these changes to bud outgrowth regulation by the R:FR ratio is not known yet. Low light intensity reduces CK contents in the nodal stem by reducing the expression of CK synthesis genes and increasing that of CK degradation genes, which inhibits bud outgrowth (solid light orange arrows and text) and up-regulates *MAX2* but the contribution of this change to bud inhibition by low light intensity is not known yet (dotted light orange arrows and text); low light intensity also decreases the sugar content, but this is not a main limiting factor in the undertaken studies. For color and arrow significations, see also **Figure 1**.

Experimental studies have revealed an interaction between light and hormonal regulators at the scale of the nodal stem segment and its bud. As illustrated in **Figure 2**, an increase in light intensity stimulates CK level in the stem, which promotes bud outgrowth, while a low R:FR ratio stimulates ABA synthesis in the bud, leading in turn to rapid bud inhibition, a process that could be reinforced by auxin signaling increase in stem. Besides these main pathways, several other endogenous regulators are impacted by light, such as the SL signaling-related genes or sugars, but their exact role has still to be understood. Evidence coming from rose under darkness or low PAR intensity indicates that stem sugars in the vicinity of the bud are not a locally limiting factor of bud outgrowth in these particular light conditions. Literature data on nodal stem segments *in vitro* rather indicate that light intensity and sugars may have a synergetic effect on bud outgrowth (Henry et al., 2011; Rabot et al., 2012; Rabot et al., 2014), as reported for the activity of the apical meristem (Li et al., 2017). This leads to the idea that the light lock should be lifted for a high sugar status of the shoot to stimulate bud outgrowth. Additional studies are also required to understand the role of elevated sugar levels in other plant parts than the stem segment bearing the bud in bud outgrowth. For example, sugars regulate nitrogen uptake by the roots (Lejay et al., 2003; Lejay et al., 2008) or hormone biosynthesis (Sakr et al., 2018), which may also indirectly impact bud outgrowth.

## Discussion

Light signaling modulates plant involvement in lateral branching by controlling the release of axillary buds from apical dominance. So far, studies mainly conducted on annuals have provided an almost complete picture of the intricate hormonal regulatory network involved in apical dominance, regardless of environmental factors (**Figure 1**). In particular, great progress has been made since the 2000s with the discovery of SL mutants and of the role of PIN proteins. The development of simulation tools made it easier to investigate complex regulations like those related to the canalization theory or to the SL molecular network, both involving feedbacks (Domagalska and Leyser, 2011). The demonstration that the degree of competition for sugars within the plant regulates bud outgrowth is more recent (Mason et al., 2014) despite first assumptions supported by computer modeling (Luquet et al., 2006; Mathieu et al., 2009). Recent evidences of interplays between sugar and hormones further complicate bud outgrowth regulating network. In addition, the main branching-related hormones display dose-dependent effects on bud outgrowth (Chatfield et al., 2000; Dun et al., 2012; Barbier et al., 2015; Corot et al., 2017) and other compounds may take a part in this mechanistic complexity, including reactive oxygen species (ROS) (Chen et al., 2016; Signorelli et al., 2018). For instance, H_2_0_2_-dependent bud outgrowth inhibition may be linked to promotion of auxin biosynthesis in the apex which inhibits CK biosynthesis in the stem in tomato (Chen et al., 2016). The presence of different regulators quantitatively regulating bud outgrowth raises the question of their integration within the bud. *BRC1* plays certainly a key role, but some regulations also occur through BRC1-independent pathways (Minakuchi et al., 2010; Braun et al., 2012; Wang et al., 2019). Recently, studies indicate that integration could be done in the regulation of carbon metabolism of the bud (Tarancon et al., 2017; Martin-Fontecha et al., 2018).

Although the major role of light intensity and quality in branching regulation has been known for decades, knowledge about the interaction between light and the endogenous regulators of bud outgrowth emerged only recently. The current knowledge (**Figure 2**) indicates that (i) light intensity stimulates production of CKs (inducer of bud outgrowth) in the nodal stem segment and (ii) a low R:FR ratio stimulates production of ABA (inhibitor of bud outgrowth) in the bud, and this process seems to be later reinforced by an increase in auxin signaling in the stem. This knowledge remains however very fragmented and does not provide a comprehensive understanding of bud outgrowth regulation at the scale of the plant, as discussed below.

First, knowledge is missing about light interaction with other endogenous regulators close to the bud. Indeed, light impacts sugar level and SL signaling (Finlayson et al., 2010; Kebrom and Mullet, 2016; Roman et al., 2016; Corot et al., 2017), which raises the question whether these different regulators act or not in the same pathway. Second, no study has addressed the question of the role of light effects on organs located at distance from buds. Light induces changes in plant growth (Granier and Tardieu, 1999; Nagel et al., 2006; Kebrom, 2017) that may alter the competition for carbohydrates within the plant and the availability of sugar for bud outgrowth. Light modulation of plant growth may also induce changes in hormone metabolism, signaling, and transport, and thereby hormone distribution and quantities. Understanding all these changes is necessary for building a comprehensive picture of light effect on bud outgrowth. Third, light regulation of bud outgrowth pattern at the scale of an axis is unknown. Light was reported to influence the number of outgrowing buds and the time between successive outgrowths (Demotes-Mainard et al., 2013; Corot et al., 2017). Future tasks would be to investigate whether light effect could result from heterogeneous distribution of the different regulators along the axis and from a temporal feedback loop by which outgrowing buds modify the regulator levels in the vicinity of the remainder buds, maintaining them dormant. However, different sensitivities of the buds to their local regulators, due to bud age, light history for example, may obviously complicate bud outgrowth regulation at axis level.

All these elements highlight the complexity of light-mediated bud outgrowth regulation at the plant scale. In recent years, the use of modeling has become prevalent to gain insight into the complex regulation of developmental processes by both endogenous and exogenous processes. These models, combining biological process description with an explicit computational description of the plant biological structure, called functional–structural plant models (FSPM), have proved meaningful to address the complexity of developmental systems as a collection of interacting constituents (at molecular or cellular level for example). FSPMs make it possible to identify and test various hypotheses on the local interaction rules and to compare qualitatively and quantitatively, with the experiments, the result emerging from these simulated interactions at an integrated level. This approach has been successfully used in the last decade to study various aspects of plant development such as flowering and inflorescence architecture development (Prusinkiewicz et al., 2007; Wenden et al., 2009), phyllotaxis (de Reuille et al., 2006; Jonsson et al., 2006; Smith et al., 2006; Refahi et al., 2016), the role of mechanics in morphogenesis (Alim et al., 2012; Boudon et al., 2015; Bozorg et al., 2016). In the study of branching regulation as well, these models have been used to help deciphering the complexity of associated regulation networks and branching processes (Evers and Vos, 2013)—for example, in the analysis of the competition for sugars (Luquet et al., 2006), auxin regulation of bud outgrowth (Prusinkiewicz et al., 2009), auxin transport in mosses (Coudert et al., 2015), and sugar interplay with auxin (Bertheloot et al., 2019). Likewise, approaches combining quantitative experimental observations and computer simulations in FSPMs are thus expected to be instrumental in providing new insights into light interplay with sugar and hormones network in bud outgrowth regulation at the plant scale. In particular, to investigate bud outgrowth regulation with FSPM, carbon/sugar fluxes formalism will have to be coupled to a formalization of hormonal functioning, as well as with a representation of the root compartment.

## Author Contributions

AS, CG, FB, SD-M, SS, and JB searched for articles, synthetized them, and wrote parts of the review; SS gave the initial impetus for writing a review around light, bud outgrowth, and plant physiology; CG and JB structured the review; JB directed the work.

## Funding

This review was conducted in the framework of a PhD thesis funded by the Environment and Agronomy department of the French National Institute for Agricultural Research (INRA), the French Region Pays de la Loire, and the program “Objectif Végétal, Research, Education and Innovation in Pays de la Loire” (supported by the Region Pays de la Loire, Angers Loire Métropole, and the European Regional Development Fund).

## Conflict of Interest

The authors declare that the research was conducted in the absence of any personal, professional, or financial relationships that could potentially be construed as a conflict of interest.
